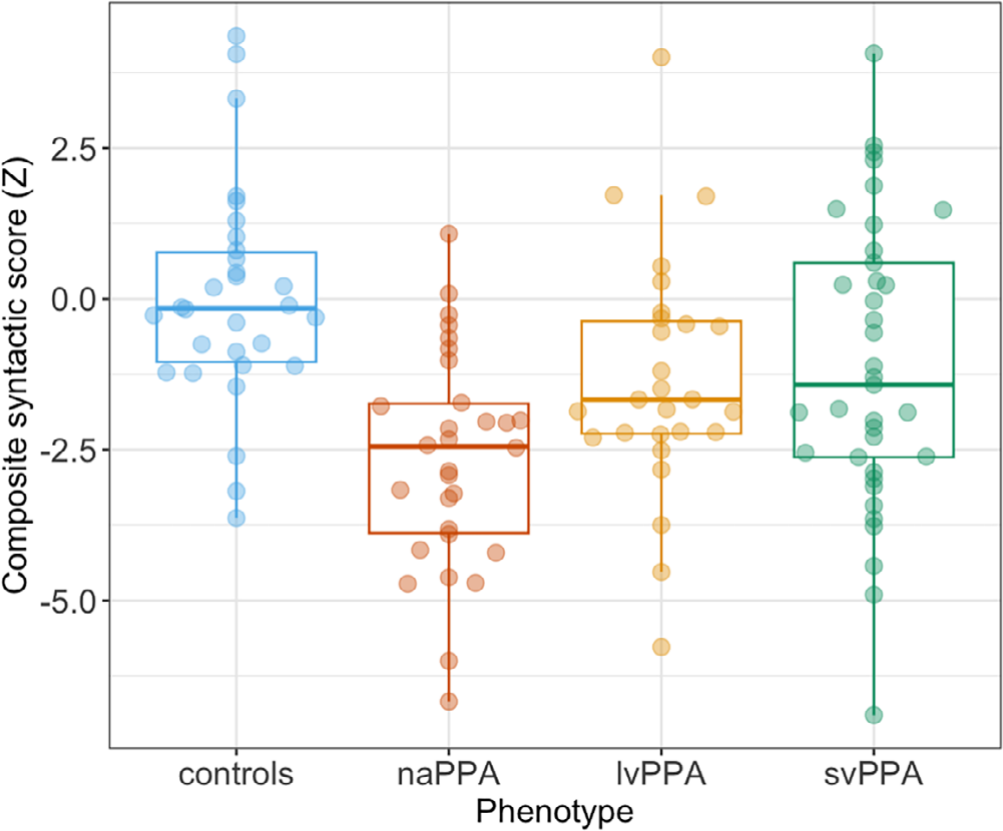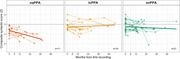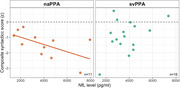# An automated method for quantifying syntactic complexity of spontaneous speech in Primary Progressive Aphasia

**DOI:** 10.1002/alz.087017

**Published:** 2025-01-09

**Authors:** Galit Agmon, Sunghye Cho, Sharon Ash, Katheryn A Q Cousins, Sameer Pradhan, Yoon Duk Kim, Mark Y Liberman, David J Irwin, Naomi Nevler

**Affiliations:** ^1^ Penn FTD Center, University of Pennsylvania, Philadelphia, PA USA; ^2^ Linguistic Data Consortium, University of Pennsylvania, Philadelphia, PA USA

## Abstract

**Background:**

Reduced syntactic complexity is the predominant linguistic impairment unique to the nonfluent agrammatic variant of PPA (naPPA).

Reliable, objective and automatic methods for assessing syntactic complexity are currently lacking. Here, we introduce an automatic method for quantifying syntactic complexity in speech. We show that this method differentiates naPPA from other subtypes and monitors disease progression and severity.

**Method:**

We analyzed speech samples of picture descriptions that were collected from PPA patients and healthy controls (HC) at the University of Pennsylvania during 2000‐2021, including a subset of patients with longitudinal data. Ten syntactic features were automatically extracted from the speech samples and combined into a composite score, using principal component analysis, to represent syntactic complexity. Regression models compared syntactic complexity between PPA phenotypes, covarying for age, sex, education and MMSE. Linear mixed‐effects models tested change in syntactic complexity over time. The correlation of syntactic complexity with neurofilament light chain (NfL) levels in the cerebrospinal fluid (CSF) was tested in naPPA and svPPA patients.

**Result:**

We examined a total of 136 participants: HC (n=36, 31% males, age 69±8y, MMSE 29±1), naPPA (n=31, 51% males, age 71±8y, MMSE 24±5), lvPPA (n=32, 50% males, age 68±9y, MMSE 22±6) and svPPA (n=37, 49% males, age 64±7y, MMSE 22±7). Z‐scored syntactic complexity scores showed significant group differences, with naPPA scoring lower than HC (β=1.08, *p*=.004), lvPPA (β=1.19, *p*=.001) and svPPA (β=.93, *p*=.008) [Fig‐1]. Sensitivity to disease progression and severity was found only within the naPPA group. The syntactic score of naPPA significantly decreased over time (‐.5 per year, *p*=.005), significantly different from the 0 decrease in lvPPA (*p*=.001) or the .1 decrease in svPPA (*p*=.001) [Fig‐2]. naPPA patients had a significant inverse correlation of syntactic complexity score and CSF NfL levels (r=‐.58, *p*=.03), while svPPA patients did not (r=.39, *p*=.9) [Fig‐3].

**Conclusion:**

We introduce a novel measure of syntactic complexity which can be extracted automatically from brief naturalistic speech samples. We validated this measure by relating it to naPPA and its biological underpinning. We provided objective evaluation of syntax, which has great potential as a non‐invasive biomarker in the dementia research and clinic.